# Orbital Pseudotumor: Uncommon Initial Presentation of IgG4-Related Disease

**DOI:** 10.1155/2015/324365

**Published:** 2015-03-09

**Authors:** Teresa Carbone, Ricardo Azêdo Montes, Beatriz Andrade, Pedro Lanzieri, Luis Mocarzel

**Affiliations:** ^1^Department of Internal Medicine, Hospital Universitário Antônio Pedro (HUAP), Universidade Federal Fluminense (UFF), Rua Marquês de Paraná 303, 7° andar, Centro, 24033-900 Niterói, RJ, Brazil; ^2^Department of Rheumatology, Hospital Universitário Antônio Pedro (HUAP), Universidade Federal Fluminense (UFF), Rua Marquês de Paraná 303, 7° andar, Centro, 24033-900 Niterói, RJ, Brazil

## Abstract

IgG4-related disease (IgG4-RD) encompasses a group of fibroinflammatory conditions recognized in recent times. The main clinical features include variable degrees of tissue fibrosis, tumorlike expansions, perivascular lymphocytic infiltration rich in IgG4-positive plasma cells, and elevated serum IgG4. A case has been reported of an elderly patient with an unexplained unilateral exophthalmia; biopsy was performed and revealed lymphocytic infiltration, suggesting IgG4-RD. High serum levels of IgG4, in association with a good response to steroid therapy and to the exclusion of other diagnoses, confirmed the hypothesis of orbital pseudotumor by IgG4-RD.

## 1. Introduction

IgG4-related disease (IgG4-RD) is a recently recognized group of fibroinflammatory conditions first described in Japan in 2001. Until now, fewer than 3,500 cases have been reported worldwide, 75% from Asia, and considered very rare in Latin America [1–3]. Many synonyms have been used to describe the same syndrome, depending on the organs involved and the country of the case in question. In 2013, a consensus was reached to suggest the name “disease related to immunoglobulin G class 4” [1, 2].

The main clinical features include variable degrees of tissue fibrosis, tumorlike expansions, perivascular lymphocytic infiltration rich in IgG4-positive plasma cells, and elevated serum IgG4 [3]. Diagnostic approach depends on clinical manifestations, IgG4 higher than the normal range, and biopsy-proven lymphoplasmacytic perivascular infiltrates with IgG4+ plasma cells, storiform fibrosis, obliterative phlebitis, and an abundance of IgG4+ plasma cells [1–5].

We report a case of an orbital pseudotumor as an unusual first manifestation of IgG4-RD.

## 2. Case Presentation

A 64-year-old white female presented to the outpatient clinic with a 3-year history of left ocular protrusion, associated with pruritus, burning sensation, and excessive tearing. She had no previous medical conditions.

On examination, there was left exophthalmo, with unilateral palpebral oedema, conjunctival hyperemia, and pain resulting from ocular globe compression. Ocular motricity was preserved, but visual acuity was reduced on the right field, with blurred vision on the right. The remainder of the physical examination was unremarkable.

Laboratory tests of thyroid function and autoantibodies were normal. Kidney function was appropriate for age, and pancreatic and hepatic enzymes were in the normal range. Search for antinuclear antibodies (ANA), anti-SSA/Ro, and ANCA showed negative results, and viral serologies (hepatitis B and hepatitis C, HIV) were negative. Immunoglobulin G class 4 was 829 mg/dL (normal: 8 to 140 mg/dL).

**Figure 1:**
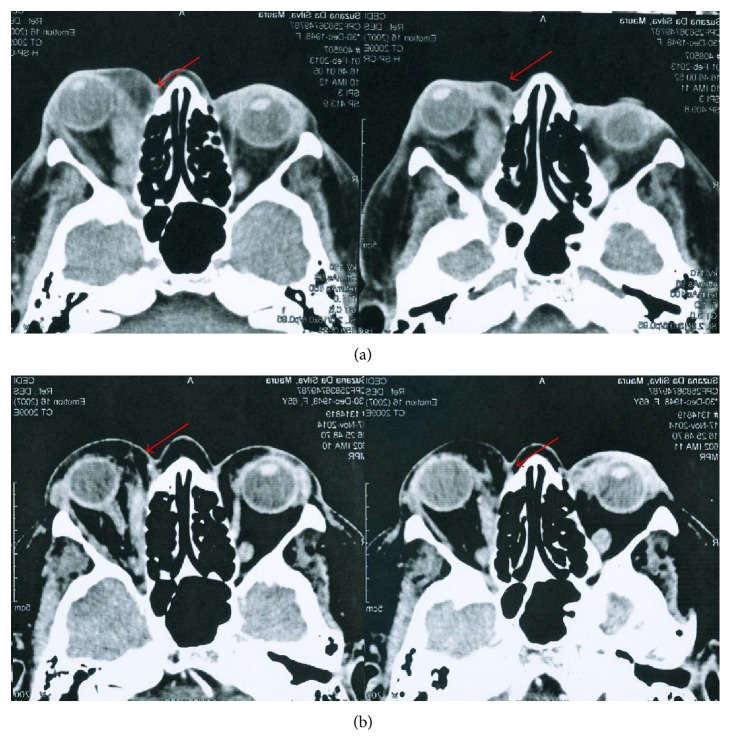
CT scan showing diffuse infiltrate of orbital tissue and muscle inflammation. Compare stages before (a) and after (b) immunosuppressive treatment.

A computed tomography (CT) scan of the head showed expansive lesion in the left orbita, with a solid mass on the retro-orbital space and contrast enhancement of rectus medial and rectus lateral of the corresponding eye (Figure 1).

**Figure 2:**
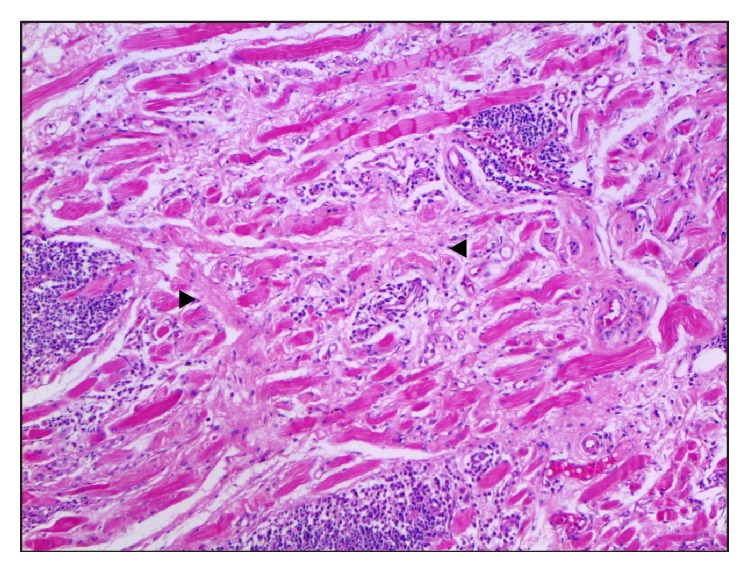
Histological sample with hematoxylin and eosin stain showing irregularly whorled pattern of fibrosis (storiform fibrosis) and lymphoplasmacytic infiltrate in the interstitium.

A biopsy was done (Figure 2) and revealed an infiltrate rich in plasma cells with increased proportion of positive IgG4 cells, positivity for CD138 antigen,* kappa* and* lambda*, with polyclonal aspect.

**Figure 3:**
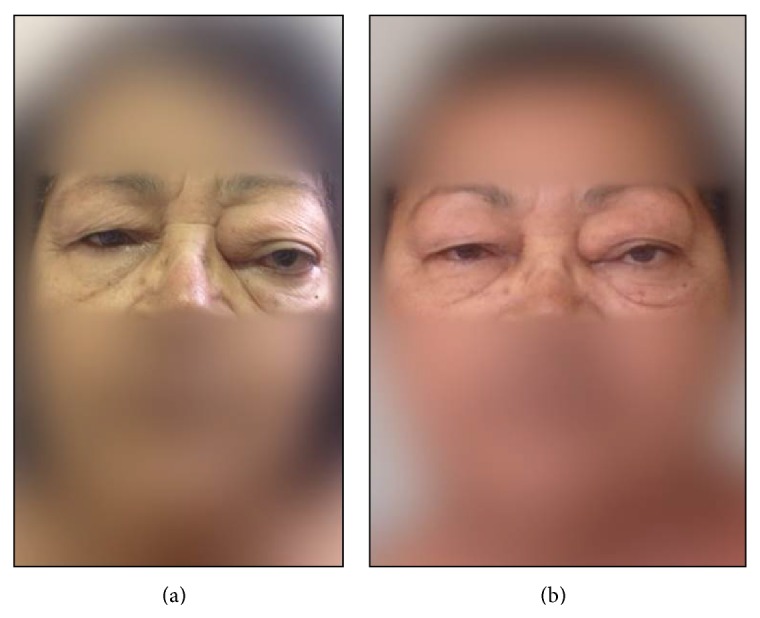
Before (a) and after (b) treatment with glucocorticoids.

As the proptosis was increasing, it was decided to start on prednisone 1 mg/Kg/day for eight weeks, followed by azathioprine 2.5 mg/Kg daily. After six months of treatment, another CT scan showed reduction in the protrusion and; after one year, the patient recovered her visual acuity and had almost no exophthalmo (Figures 1 and 3).

## 3. Discussion

IgG4-RD is a recently recognized syndrome with many unexplained aspects, but with specific serological, clinical, and histological features. It generally presents with a unique organ infiltration and is frequently misdiagnosed as malignancy [5]. Many reports of inflammatory pseudotumor in different sites may be examples of undiagnosed IgG4-RD [6, 7].

The first case reported was described by Hamano et al.: several young patients with pancreatitis with no clear etiology and high serum levels of IgG4 ended up developing pancreas sclerosis [1]. Since that, more than forty organ involvements were suggested, about three-fourths of that in Japanese patients [5].

Clinical aspects are variable and are more often described in old males in their sixth decade of life: sclerosing cholangitis, glomerulopathies, dacryoadenitis and sclerosing sialadenitis, idiopathic fibrosis (retroperitoneal, mediastinal, and thyroid), pulmonary inflammatory pseudotumors and interstitial pneumonitis, pachymeningitis, and hypophysitis [4–7]. The presentation is often subclinical: pseudotumors and fibrosis are the most evident clinical clues and may be associated with systemic manifestations, such as weight loss and change in bowel movements [1, 6]. In particular in Latin America, it is usually suspected after the histopathological results indicating “chronic inflammatory processes” in tissue specimens, making it harder to estimate its prevalence.

On complementary methods, serum levels of IgG4 higher than 135 mg/dL used to be a diagnostic criterion for IgG4-RD. However, initial stages of the disease, or a patient with immunodeficiency, may present with normal levels of the biomarker, in which it is preferable to use the relation of serum IgG4/total IgG higher than 8% as a high sensitive and specific criterion [7]. Histopathological diagnosis of IgG4-RD is defined when there is a relation of IgG4+/IgG+ total on plasmocytes higher than 40%, with a specificity of more than 85%. In case of storiform fibrosis and obliterative phlebitis, the specificity goes up to 100% but decreases the sensitivity to less than 50% of cases, which supports the need of more data to define diagnostic criteria [8].

Lacrimal and orbital involvements are rare and are called IgG4-related ophthalmic disease, frequently presenting as orbital myositis, perineuritis of the optic and trigeminal nerves, orbital inflammation, or scleritis [7]. Most common clinical presentations are unilateral or bilateral swelling of eyelids and orbita, and 30% of cases may present with proptosis, as happened in the case reported [9, 10]. Imaging findings in CT scan or magnetic resonance are not specific, making it difficult to differentiate between inflammatory patterns due to granulomatosis with polyangiitis (Wegener), which classically presents with sinusopathy [11]. Definitive differential diagnosis may rely on histopathology and, as in this case, the response to treatment was also a clue and could be documented by reduction of proptosis in CT images. As the patient in this case did not have any other organ involvements, such as chronic sclerosing sialadenitis, type 1 autoimmune pancreatitis, or tubulointerstitial nephritis, which would be typical and frequent in IgG4-related disease, the diagnosis of IgG4-RD was supported using histological analysis with IgG4-immunostaining (typically storiform fibrosis).

Since the first clinical feature presented in this case was orbital pseudotumor, the most common differential diagnosis had to be excluded. As the thyroid exam was normal and the extraocular muscles were preserved on the CT scan (in accordance with what is expected in IgG4-RD), exophthalmos due to Graves' disease was less probable [11]. Sjögren's syndrome (SS) may present with abnormal values of IgG4 in a minority of cases, with mean value lower (around 23 mg/dL) than that in patients with IgG4-RD (697 mg/dL), the histopathological finding is a lymphocytic infiltrate, and the response to glucocorticoids is poor [5, 12]. Since there were no symptoms suggestive of SS and the autoantibodies tests were negative, this was also a less probable diagnosis. Tolosa-Hunt disease is a rare condition in which the ocular manifestations are common, there are no serum markers, and the definitive diagnostic is made by biopsy showing granulomatous infiltrate around sagittal veins and other diseases have been excluded [13, 14].

Glucocorticoids are the first-line therapy to IgG4-RD, regardless of the organ involvement [1, 4, 6, 9]. The expected response to therapy is a symptom relief, which depends on organ volume decrease and, generally, is followed by reduction in serum IgG4 levels [9]. Distinctively, reactivation of the disease may be accompanied by elevations of serum IgG, and the response to glucocorticoid can be monitored by decreases in the dosage [1, 6].

Azathioprine, methotrexate, and mycophenolate are used with limited success in patients with advanced IgG4-RD and corticosteroid dependency and, in these cases, the use of rituximab may be beneficial [9]. In cases of corticosteroid resistance, it is always important to reassess the diagnosis, since it has been shown that many apparent refractory patients were misdiagnosed, which reinforces the need of more definite diagnostic criteria [7]. Making the correct diagnosis is important as the disease is usually steroid responsive although relapse rates are high [10, 11].

Recently, an association of B cell lymphoma with IgG4-RD [14] has been described. The pathogenesis of IgG4-RD is poorly understood, but it is hypothesized that the chronic inflammation may correlate with hematologic neoplasms. Since this patient had no hematological alterations in blood tests, it was decided only to follow up with no additional investigation.

The relevance in reporting IgG4-RD cases, especially in unusual scenarios, such as the orbital pseudotumor, remains an important contribution to the many possible disease presentations, as this is still an underdiagnosed condition in which the treatment may lead to remission and prevent significant morbidity and mortality. Future studies should focus on identifying biomarkers to assist with noninvasive methods of diagnosis and testing novel treatments that might supplant glucocorticoids as first line agents of treatment, providing better responses or, at least, requiring minimal doses of steroids. We believe that anti-B cells therapies (such as rituximab or belimumab) may be promising choices, as the disease probably relies on B-lymphocytes to progress.
